# Enlargement of perivascular spaces associated with habitual sake (Japanese rice wine) consumption in participants of brain health checkups

**DOI:** 10.1007/s00234-026-03917-w

**Published:** 2026-02-06

**Authors:** Naoki Omori, Fusao Ikawa, Masaaki Chiku, Ryuji Kawata, Shuhei Yamaguchi, Atsushi Nagai

**Affiliations:** 1 Shimane University, Izumo, Japan; 2 Shimane Prefectural Central Hospital, Izumo, Japan; 3 Medical Check Studio Tokyo Ginza Clinic, Tokyo, Japan; 4 EUCALIA, Inc, Tokyo, Japan; 5 Hospital Bureau of Shimane Prefecture, Matsue, Japan

**Keywords:** Alcohol-related problems, Cerebral small vessel disease, Arteriosclerosis, MRI

## Abstract

**Background:**

The prevalence of cerebral small vessel disease (SVD) among individuals who consume moderate amounts of alcohol remains unclear. This study aimed to evaluate the association between alcohol consumption and the severity of SVD markers.

**Method:**

This cross-sectional study included participants who underwent brain health checkups at a single facility. Drinkers were categorized as occasional (three or fewer times per week) and frequent (four or more times per week). Alcohol intake was assessed in units of one glass of sake, a traditional Japanese alcoholic beverage. The severity of periventricular hyperintensity (PVH), deep subcortical white matter hyperintensity (DWMH), cerebral microbleeds (CMB), and enlargement of perivascular spaces in the basal ganglia (PVS-BG) and perivascular spaces in the centrum semiovale (PVS-CSO) were evaluated as SVD indicators.

**Results:**

A total of 64,659 participants were included in this study. In multivariable ordinal logistic regression analysis, frequent drinkers had higher odds of PVS-BG enlargement (odds ratio [OR] = 1.09, 95% confidence interval [CI]: 1.02–1.15, *p* = 0.006) and PVS-CSO (OR = 1.23, 95% CI: 1.16–1.31, *p* < 0.001) compared with non-current drinkers. Occasional drinkers also had higher odds of PVS-CSO enlargement (OR = 1.08, 95% CI: 1.02–1.14, *p* = 0.007). Moreover, the consumption of one glass of sake per day (equivalent to 20 g of pure alcohol) was associated with greater severity of PVS-BG and PVS-CSO enlargement.

**Conclusions:**

PVS enlargement, particularly PVS-CSO, showed a moderate association with habitual alcohol consumption. These findings suggest that PVS may serve as a potential imaging biomarker reflecting subtle structural brain alterations associated with alcohol exposure.

**Supplementary Information:**

The online version contains supplementary material available at 10.1007/s00234-026-03917-w.

## Introduction

Health risks associated with excessive alcohol consumption remain a major public health concern worldwide. Habitual alcohol consumption is carcinogenic, increasing the risk of esophageal and colorectal cancers, and is a well-established risk factor for cardiovascular diseases such as hypertension and heart failure [1, 2]. Chronic alcohol exposure can also cause severe damage to the central nervous system (CNS) [3–5]. For example, several prospective studies have suggested that long-term alcohol consumption increases the risk of developing dementia [6, 7]. Additionally, excessive alcohol intake, typically defined as consumption exceeding approximately 60 g of pure alcohol per day, has been reported to increase the risk of stroke [8].

Cerebral small vessel disease (SVD) refers to the pathological changes caused by damage to small blood vessels in the brain, which can be detected using magnetic resonance imaging (MRI). Imaging findings associated with SVD include asymptomatic abnormalities such as white matter lesion (WML), cerebral microbleeds (CMB), and perivascular spaces (PVS) enlargement. These SVD markers have recently gained attention as potential biomarkers for predicting future cerebrovascular disease and dementia [9–12]. Previous studies have reported a significant increase in asymptomatic MRI findings associated with SVD among individuals with alcohol dependence [13, 14].

However, the prevalence of SVD among individuals who consume alcohol within a moderate or so-called “healthy” range remains unclear. Moreover, the threshold at which alcohol consumption becomes a risk factor for SVD remains to be determined. In Japan, brain health checkups, commonly known as Brain Dock, are widely performed to assess asymptomatic brain lesions and atrophy related to cerebrovascular disease and dementia [15]. This study aimed to investigate alcohol consumption habits among individuals who underwent Brain Dock assessments and evaluate the association between alcohol intake and SVD markers.

## Methods

### Participants and inclusion criteria

**Fig. 1:**
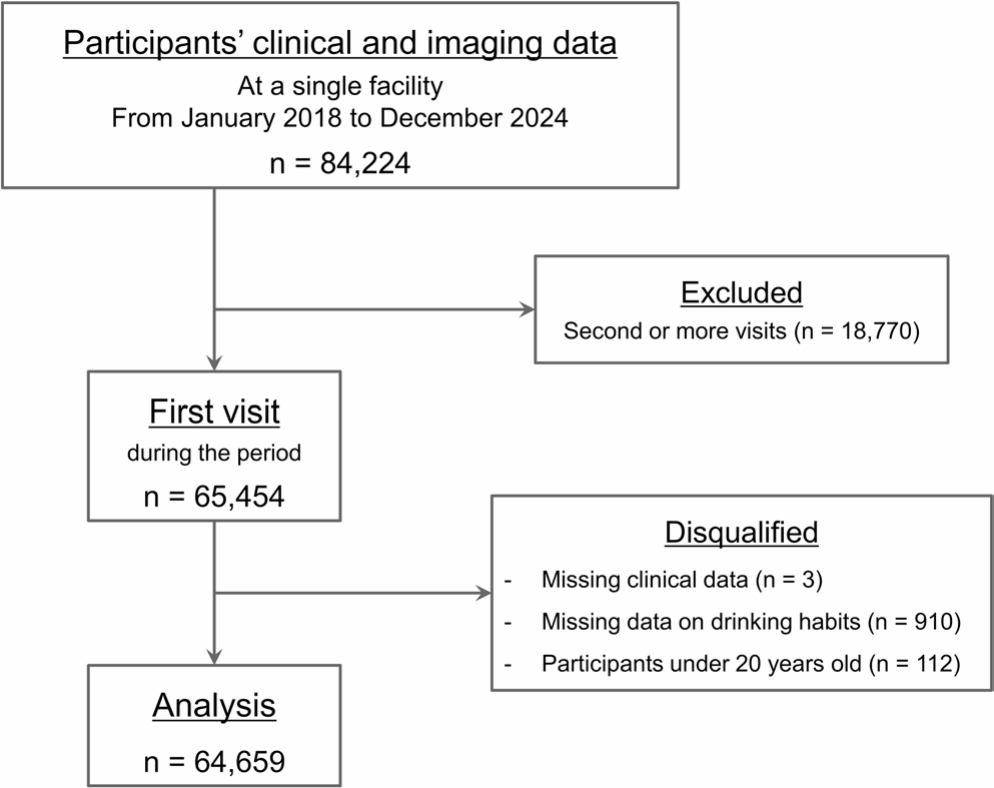
Flowchart of study sample selection

The Brain Dock assessments using MRI and magnetic resonance angiography have been offered for brain health checkups in Japan since 1995 [15]. In addition to MRI, data were collected on participants’ medical histories, drinking and smoking habits, and physical examinations. The Brain Dock is a Japanese health checkup service that stores imaging data from collaborating facilities in cloud storage and promptly notifies examinees of diagnoses by expert readers. We obtained data from participants who underwent brain health checkups at a single facility (Medical Check Studio Tokyo Ginza Clinic, Tokyo, Japan) between January 2018 and December 2024. For participants with multiple visits during the study period, only the data from the first visit were included in the analysis. Participants who did not answer questions about their alcohol drinking habits and those younger than 20 years were excluded from the analysis (Fig. 1). No exclusion was made based on the presence of underlying medical conditions, neurological disorders, or prior cerebrovascular disease, as the study aimed to reflect real-world brain health checkup participants.

### Clinical assessment

Participants who underwent the Brain Dock assessment were instructed to provide information on their age (years), sex (male/female), body mass index (BMI), smoking habits, medical histories (hypertension, diabetes mellitus, and dyslipidemia), and alcohol consumption habits, including frequency and amount consumed (see Supplementary Materials for further details). BMI was measured as the ratio of the participants’ weight to the square of their height (kg/m^2^). Smoking habits were classified into four categories: nonsmokers, current smokers, former smokers, and those exposed to secondhand smoke. Medical histories were obtained using a self-administered questionnaire. Hypertension, diabetes mellitus, and dyslipidemia were considered present if the participant had been diagnosed with the condition by a medical professional, regardless of current treatment status. Alcohol consumption habits were categorized as follows: non-current drinkers (those who did not currently consume alcohol), occasional drinkers (those who consumed alcohol regularly but no more than three times per week), and frequent drinkers (those who consumed alcohol four or more times per week). Information on past drinking history was not collected in this study; therefore, former drinkers were classified as non-current drinkers. Alcohol intake was assessed based on the standard unit of one glass of sake (Japanese rice wine), which is one of the most commonly consumed alcoholic beverage in Japan. One glass of sake (180mL) contains approximately 15% ethanol, equivalent to 20 g of pure alcohol [16]. Using this standardized unit helped reduce the variability in alcohol intake estimates. However, participants who primarily consumed beer or wine were unable to accurately convert their intake into sake units and therefore did not provide responses regarding alcohol consumption volume. Participants who reported their alcohol consumption in sake units were categorized by daily intake as follows: 1 glass or less, 1–2 glasses, 2–3 glasses, and > 3 glasses per day.

### Neuroimaging

MRI was performed on the same day as the clinical assessment using a 1.5 T scanner (ELAN, Canon Medical Systems, Tochigi, Japan). The protocol included conventional T1-weighted, T2-weighted, fluid-attenuated inversion recovery, and T2*-weighted imaging. The acquisition parameters are listed in Supplementary Table 1. The scans were double-checked by skilled neuroradiologists and neurosurgeons who were blinded to the participants’ clinical information.

We focused on WML, CMB, and PVS to assess the severity of SVD in accordance with the STRIVE-2 statement [10]. WML was evaluated separately as periventricular hyperintensity (PVH) and deep subcortical white matter hyperintensity (DWMH), and the classification was based on previous criteria [17, 18]. PVH was classified as follows: grade 0, absent or “periventricular rims” only; grade I, localized lesion “cap” or pencil-thin lining; grade II, extended along the entire periventricular area; grade III, irregular PVH extending into the deep white matter; or grade IV, extending throughout the deep and subcortical white matter. DWMH was classified as follows: grade 0, absent; grade I, punctate foci, diameter ≤ 3 mm, boundary clear; grade II, diameter ≥ 3 mm, punctate or discrete foci; grade III, large confluent foci, boundary unclear; or grade IV, confluence widely distributed in most of the white matter. CMB are small (generally 2–5 mm in diameter) areas of signal void with blooming artifacts on T2*-weighted MRI, classified according to lesion number as follows: grade 0, none; grade I, counts 1; grade II, counts 2–5; grade III, counts 6–10; or grade IV, counts ≥ 11. PVS referred to ovoid or linear lesions that were visible as hypointense and hyperintense regions in the basal ganglia (PVS-BG) or centrum semiovale (PVS-CSO) on T1- and T2-weighted images, respectively, and were considered “enlarged” if their size was ≥ 2 mm. The guidelines of the Japanese Brain Dock Society established a classification based on the number of PVS observed at the basal ganglia or centrum semiovale level [19]. PVS-BG was classified as follows: grade 0, none; grade I, counts 1–5; grade II, counts 6–10; or grade III, counts ≥ 11. PVS-CSO was classified as follows: grade 0, none; grade I, counts 1–10; grade II, counts 11–20; grade III, counts 21–40; or grade IV, counts ≥ 41. Although old lacunes are recognized as an important marker of SVD, they were not evaluated in the present study because their prevalence was extremely low in this health checkup–based cohort, making reliable grading difficult. Further details on the diagnostic imaging procedures are described in the Supplementary Materials.

### Selection of covariates

Our study controlled for several demographic covariates, including age, sex, and BMI. Smoking habits were also included as covariates, as they are not only a risk factor for cerebrovascular disease but may also be associated with the loss of structural integrity in the white matter and the severity of PVS [20, 21]. Furthermore, hypertension, diabetes mellitus, and dyslipidemia are known risk factors for the progression of chronic ischemic change and microhemorrhage [22, 23]. Hypertension has also been suggested to be associated with the severity of PVS in the basal ganglia [24, 25]. Therefore, these variables were included as covariates in our analysis, and multivariable analysis was performed.

### Statistical analysis

Descriptive statistics were used to summarize clinical characteristics, medical histories, and imaging data, including means, standard deviations, and percentages. Each variable was stratified into three groups: non-current drinkers, occasional drinkers, and frequent drinkers. Categorical variables were compared using Fisher’s exact test, and continuous variables were analyzed using the Kruskal–Wallis test.

The association between alcohol consumption categories and the severity of SVD markers was examined using ordinal logistic regression models. Each marker (grade I–III or 0–IV) was treated as an ordinal outcome. In the univariable ordinal logistic regression analysis (Model 1), alcohol consumption was entered as a categorical variable (non-current drinkers, occasional drinkers, and frequent drinkers), and odds ratios (ORs) with 95% confidence intervals (CIs) were calculated using non-current drinkers as the reference group. In the multivariable analyses (Model 2), age, sex, BMI, smoking habits, and histories of hypertension, diabetes mellitus, and dyslipidemia were included as covariates, and the adjusted ORs were calculated. Model assumptions were assessed using the variance inflation factor (VIF) and the Brant test. VIF values greater than 5 were considered indicative of multicollinearity. The Brant test was used to verify the proportional odds assumption, and non-significant results (*p* > 0.05) were interpreted as evidence that the assumption was satisfied.

Subgroup analyses were conducted to explore potential effect modification by age, sex, and BMI. Participants were classified into subgroups based on age (20–40, 41–60, and > 60 years), sex (male/female), and BMI according to Japanese criteria (< 18.5, 18.5–25, and ≥ 25 kg/m^2^) [26]. For each SVD markers, interaction tests were conducted across strata. Subsequently, alcohol consumption was categorized by units of sake consumed per day, and stratified ORs and 95% CIs were calculated for each consumption group. To adjust for multiple testing, Bonferroni correction was applied: a correction factor of 3 was used for interaction tests (age, sex, and BMI), and a factor of 4 was applied for comparisons among the four drinking categories (excluding the reference group). Adjusted *p* values were calculated by multiplying the original *p* values by the corresponding correction factors.

All analyses were performed using EZR (Saitama Medical Center, Jichi Medical University, Saitama, Japan), a graphical user interface for R (The R Foundation for Statistical Computing, Vienna, Austria, version 4.3.1) [27]. More precisely, it is a modified version of R Commander (version 2.9-1.9.9) designed to add statistical functions frequently used in biostatistics. Statistical significance was defined as a two-sided *p* value < 0.05.

## Results

### Characteristics of the participants

**Fig. 2:**
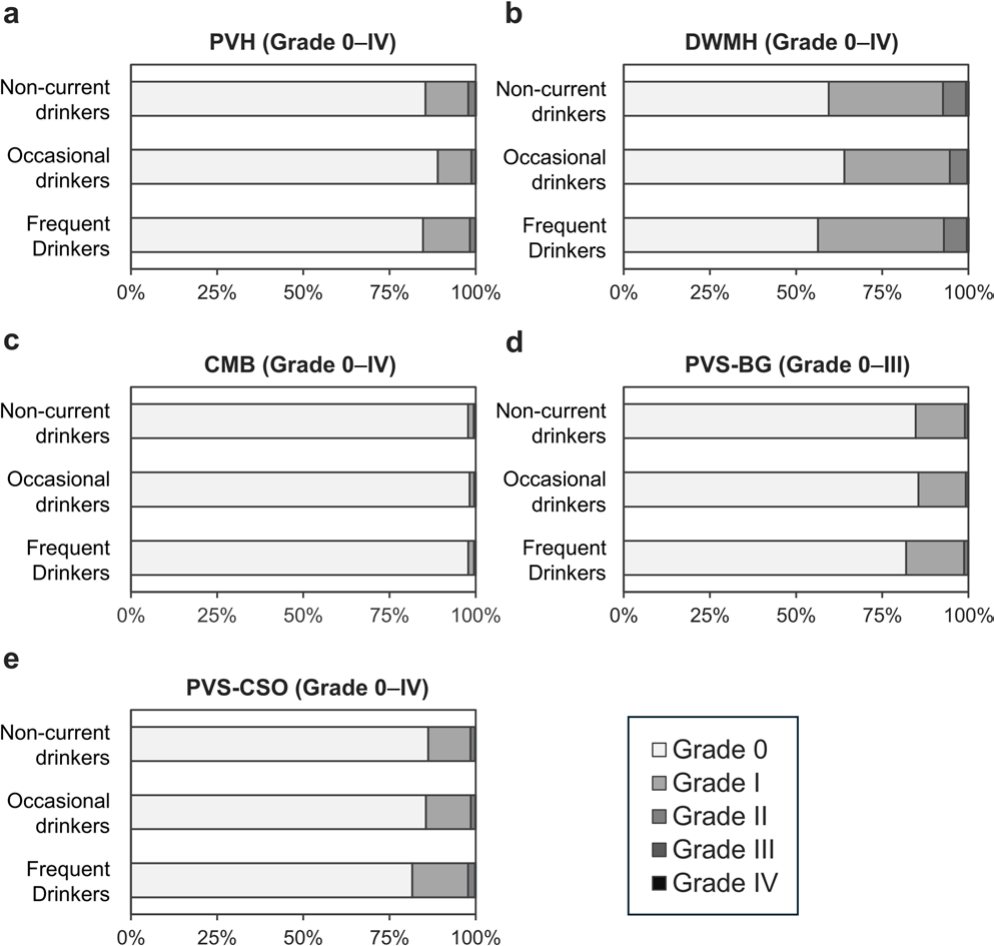
Bar chart showing the percentage of severity of cerebral small vessel diseases. The severity was classified from grades 0 to IV for PVH (**a**), DWMH (**b**), CMB (**c**), and PVS-CSO (**e**). For the PVS-BG (**d**), the severity was classified from grades 0 to III. The detailed percentages are shown in the Supplementary Materials. PVH, periventricular hyperintensity; DWMH, deep subcortical white matter hyperintensity; CMB, cerebral microbleed; PVS, perivascular space; BG, basal ganglia; CSO, centrum semiovale

The baseline characteristics of the study participants are presented in Table 1. A total of 64,659 participants were included in the analysis, with a mean age of 49.28 years; 53.6% were men, and 46.4% were women. The mean ages of non-current drinkers, occasional drinkers, and frequent drinkers were 49.96, 47.30, and 51.21 years, respectively, with significant differences among the groups (*p* < 0.001). Non-current drinkers were more likely to be female, whereas occasional and frequent drinkers were more likely to be male (*p* < 0.001). The overall mean BMI was 23.06 kg/m^2^ and was slightly lower among non-current drinkers than among occasional and frequent drinkers (*p* < 0.001). Significant differences were also observed among the groups in the proportion of current and former smokers and in the prevalence of hypertension, diabetes, and dyslipidemia (all *p* < 0.001). Among the participants who regularly consumed sake and reported their alcohol intake, 9,559 were occasional drinkers and 7,862 were frequent drinkers, accounting for approximately 40% of all drinkers. Most occasional drinkers reported consuming one or fewer glasses per day, whereas most frequent drinkers reported consuming one to two glasses per day. The MRI images of all participants were reviewed by experts to assess the severity of PVH, DWMH, CMB, PVS-BG, and PVS-CSO (Fig. 2a–e and Supplementary Table 2). More than half of the participants had no detectable lesions (grade 0) in any of these regions.

**Table 1:** Baseline characteristics of the participants stratified based on alcohol consumption habits

	Total	Non-current drinkers	Occasional drinkers	Frequent drinkers	*p* value
	*n* = 64,659	*n* = 19,671	*n* = 25,582	*n* = 19,406	
Age, mean (SD), year	49.28 (11.07)	49.96 (11.79)	47.30 (10.93)	51.21 (10.01)	< 0.001
20–40, n (%)	13,584 (21.0)	4,052 (20.6)	6,851 (26.8)	2,681 (13.8)	
41–60, n (%)	41,220 (63.7)	12,085 (61.4)	15,870 (62.0)	13.265 (68.4)	
> 60, n (%)	9,855 (15.2)	3,534 (18.0)	2,861 (11.2)	3,460 (17.8)	
Sex					
Men, n (%)	34,641 (53.6)	7,735 (39.3)	13,645 (53.3)	13,261 (68.3)	< 0.001
BMI, mean (SD), kg/m^2^	23.06 (3.55)	22.85 (3.77)	23.13 (3.64)	23.18 (3.19)	< 0.001
< 18.5, n (%)	4,201 (6.5)	1,714 (8.7)	1,529 (6.0)	958 (4.9)	
18.5–25, n (%)	44,456 (68.8)	13,307 (67.6)	17,538 (68.6)	13,621 (70.2)	
> 25, n (%)	15,992 (24.7)	4,650 (23.6)	6,515 (25.5)	4,827 (24.9)	
Smoking habits					< 0.001
Nonsmoker, n (%)	42,118 (65.1)	14,979 (76.1)	17,565 (68.7)	9,574 (49.3)	
Former smoker, n (%)	8,865 (13.7)	1,915 (9.7)	3,088 (12.1)	3,862 (19.9)	
Current smoker, n (%)	13,365 (20.7)	2,631 (13.4)	4,817 (18.8)	5,917 (30.5)	
Exposure to secondhand smoke	311 (0.5)	146 (0.7)	112 (0.4)	53 (0.3)	
Medical histories					
Hypertension, n (%)	12,941 (20.0)	3,569 (18.1)	4,215 (16.5)	5,157 (26.6)	< 0.001
Diabetes mellitus, n (%)	2,605 (4.0)	878 (4.5)	973 (3.8)	754 (3.9)	< 0.001
Dyslipidemia, n (%)	11,385 (17.6)	3,923 (19.9)	4,399 (17.2)	3,063 (15.8)	< 0.001
Sake consumption per day					
Not drinking sake, n (%)	NA	NA	15,723 (61.5)	11,544 (59.5)	
Drinking sake, n (%)	NA	NA	9,859 (38.5)	7,862 (40.5)	
< 1 glass per day, n (%)	NA	NA	5,216 (20.4)	1,671 (8.6)	
1–2 glasses per day, n (%)	NA	NA	3,075 (11.9)	3,418 (17.6)	
2–3 glasses per day, n (%)	NA	NA	1,033 (4.0)	1,853 (9.5)	
> 3 glasses per day, n (%)	NA	NA	535 (2.1)	920 (4.7)	

Values represent means (standard deviations) for continuous variables and n (%) for categorical variables. The Kruskal–Wallis test was used to compare age and BMI. Fisher’s exact test was used to compare group differences in sex, smoking habits, and histories of hypertension, diabetes mellitus, and dyslipidemia. Sake is a traditional Japanese alcoholic beverage, containing approximately 20 g of ethanol in 180 mL per glass

*SD* standard deviation, *BMI* body mass index

### Association between alcohol consumption habits and cerebral SVD markers and subgroup analysis

We performed ordinal logistic regression analyses to evaluate the association between alcohol consumption habits and the severity of SVD markers, using non-current drinkers as the reference group. In the univariable analyses (Model 1), occasional drinkers had significantly lower odds of PVH (OR = 0.72, 95% CI: 0.68–0.76, *p* < 0.001), DWMH (OR = 0.81, 95% CI: 0.78–0.84, *p* < 0.001), CMB (OR = 0.83, 95% CI: 0.72–0.94, *p* = 0.005), and PVS-BG (OR = 0.93, 95% CI: 0.88–0.98, *p* = 0.005) compared with non-current drinkers. Conversely, frequent drinkers had significantly higher odds of DWMH (OR = 1.11, 95% CI: 1.06–1.15, *p* < 0.001), PVS-BG (OR = 1.21, 95% CI: 1.15–1.28, *p* < 0.001), and PVS-CSO (OR = 1.41, 95% CI: 1.33–1.49, *p* < 0.001) (Table 2). However, in multivariable analyses adjusted for covariates (Model 2), occasional drinkers showed significantly higher odds only for PVS-CSO (adjusted OR = 1.08, 95% CI: 1.02–1.14, *p* = 0.007). Frequent drinkers remained at increased odds for both PVS-BG (adjusted OR = 1.09, 95% CI: 1.02–1.15, *p* = 0.006) and PVS-CSO (adjusted OR = 1.23, 95% CI: 1.16–1.31, *p* < 0.001). The VIF of all variables was less than 5 in each model, indicating that there was no multicollinearity among the variables. The proportional odds assumption was evaluated using the Brant test. Alcohol consumption satisfied this assumption in most models; however, in the model for DWMH, the assumption was violated. In addition, several covariates, including age and hypertension, also did not meet the assumption in some models. These violations suggest that the effects of these variables may vary across outcome levels. Therefore, caution is warranted when interpreting the regression results (Supplementary Table 3).

**Table 2:** Univariable and multivariable logistic regression analyses of alcohol consumption habits for the presence of small vessel diseases

	PVH		DWMH		CMB		PVS-BG		PVS-CSO	
	OR (95% CI)	*p* value	OR (95% CI)	*p* value	OR (95% CI)	*p* value	OR (95% CI)	*p* value	OR (95% CI)	*p* value
Model 1 (not adjusted)										
Non-current drinker	1 (ref)		1 (ref)		1 (ref)		1 (ref)		1 (ref)	
Occasional drinker	0.72 (0.68–0.76)	**< 0.001**	0.81 (0.78–0.84)	**< 0.001**	0.83 (0.72–0.94)	**0.005**	0.93 (0.88–0.98)	**0.005**	1.05 (0.99–1.10)	0.010
Frequent drinker	1.05 (0.99–1.11)	0.108	1.11 (1.06–1.15)	**< 0.001**	1.04 (0.91–1.19)	0.575	1.21 (1.15–1.28)	**< 0.001**	1.41 (1.33–1.49)	**< 0.001**
Model 2 (adjusted for covariates)										
Non-current drinker	1 (ref)		1 (ref)		1 (ref)		1 (ref)		1 (ref)	
Occasional drinker	1.02 (0.95–1.08)	0.643	1.03 (0.98–1.07)	0.185	0.98 (0.85–1.14)	0.800	1.01 (0.96–1.07)	0.705	1.08 (1.02–1.14)	**0.007**
Frequent drinker	1.05 (0.98–1.12)	0.166	1.03 (0.98–1.07)	0.290	0.89 (0.77–1.04)	0.141	1.09 (1.02–1.15)	**0.006**	1.23 (1.16–1.31)	**< 0.001**

Model 1 was not adjusted for covariates

Model 2 was adjusted for age, sex, body mass index, smoking habits, and a history of hypertension, diabetes mellitus, and dyslipidemia. Bold type indicates a significant association in the 95% CI

*PVH* periventricular hyperintensity, *DWMH* deep subcortical white matter hyperintensity, *CMB* cerebral microbleed, *PVS* perivascular space, *BG* basal ganglia, *CSO* centrum semiovale, *OR* odds ratio, *CI* confidence interval, *ref* reference

**Fig. 3:**
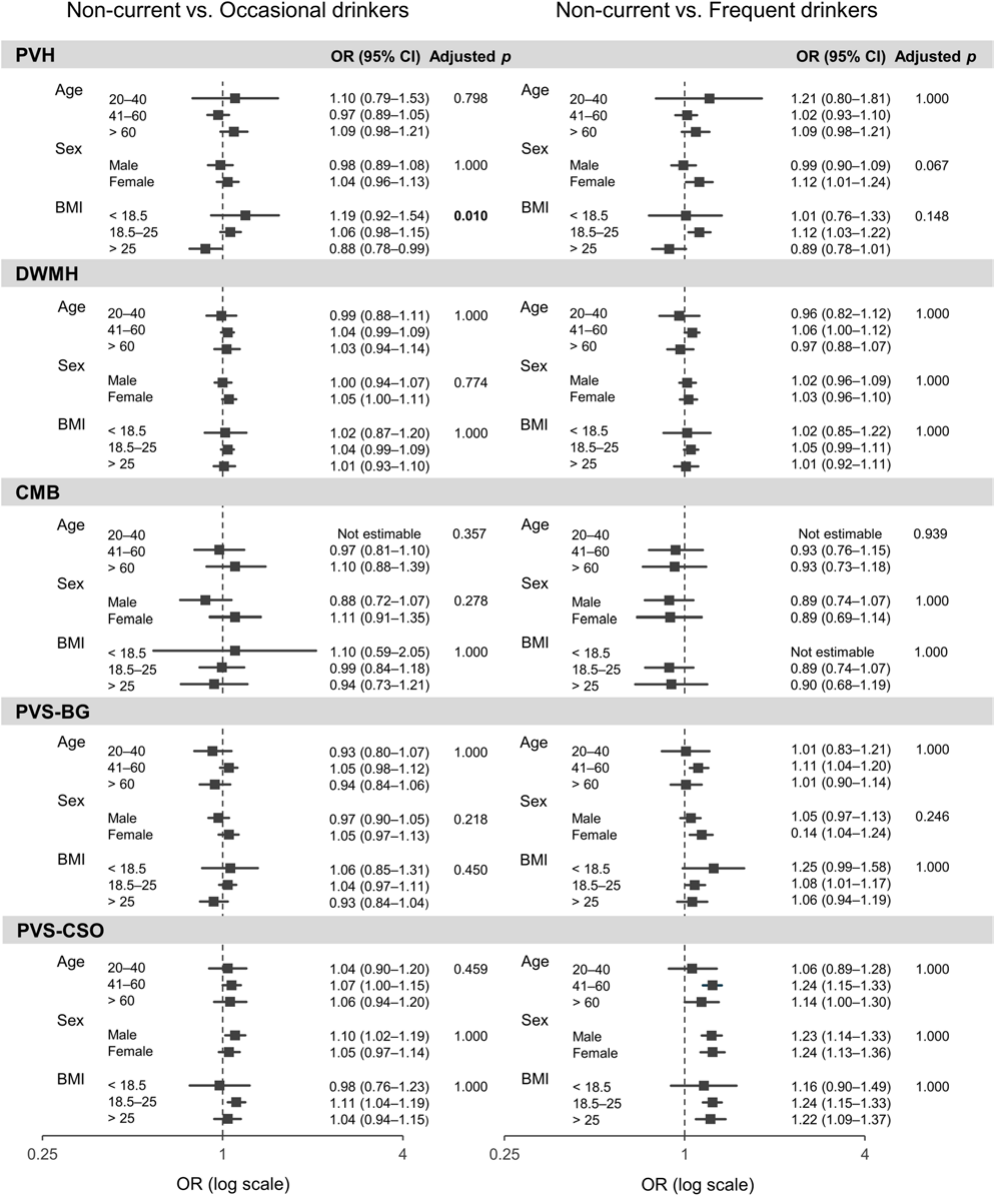
Forest plot summarizing the multivariable ordinal logistic regression analyses performed for each age, sex, and BMI subgroup. All models were adjusted for age, sex, BMI, smoking habits, and histories of hypertension, diabetes mellitus, and dyslipidemia. Bold values indicate significant interactions between the subgroups after correction for multiple comparisons. PVH, periventricular hyperintensity; DWMH, deep subcortical white matter hyperintensity; CMB, cerebral microbleed; PVS, perivascular space; BG, basal ganglia; CSO, centrum semiovale; OR, odds ratio; CI, confidence interval; BMI, body mass index

Subsequently, exploratory subgroup analyses stratified based on age, sex, and BMI were performed to examine potential effect modification (Fig. 3). Although several interaction terms were initially significant in unadjusted analyses, only the interaction between BMI and alcohol consumption (non-current drinkers vs. occasional drinkers) on PVH severity remained statistically significant after Bonferroni correction (adjusted *p* < 0.05). In this group, individuals with a BMI ≥ 25 kg/m² exhibited a lower odds ratio compared with those in lower BMI categories. Other interactions did not remain significant after correction for multiple comparisons. In certain subgroups, odds ratios could not be estimated because of sparse data or zero events within specific strata (indicated as “Not estimable” in Fig. 3).

### Association between the amount of alcohol consumption and cerebral SVD markers

**Fig. 4:**
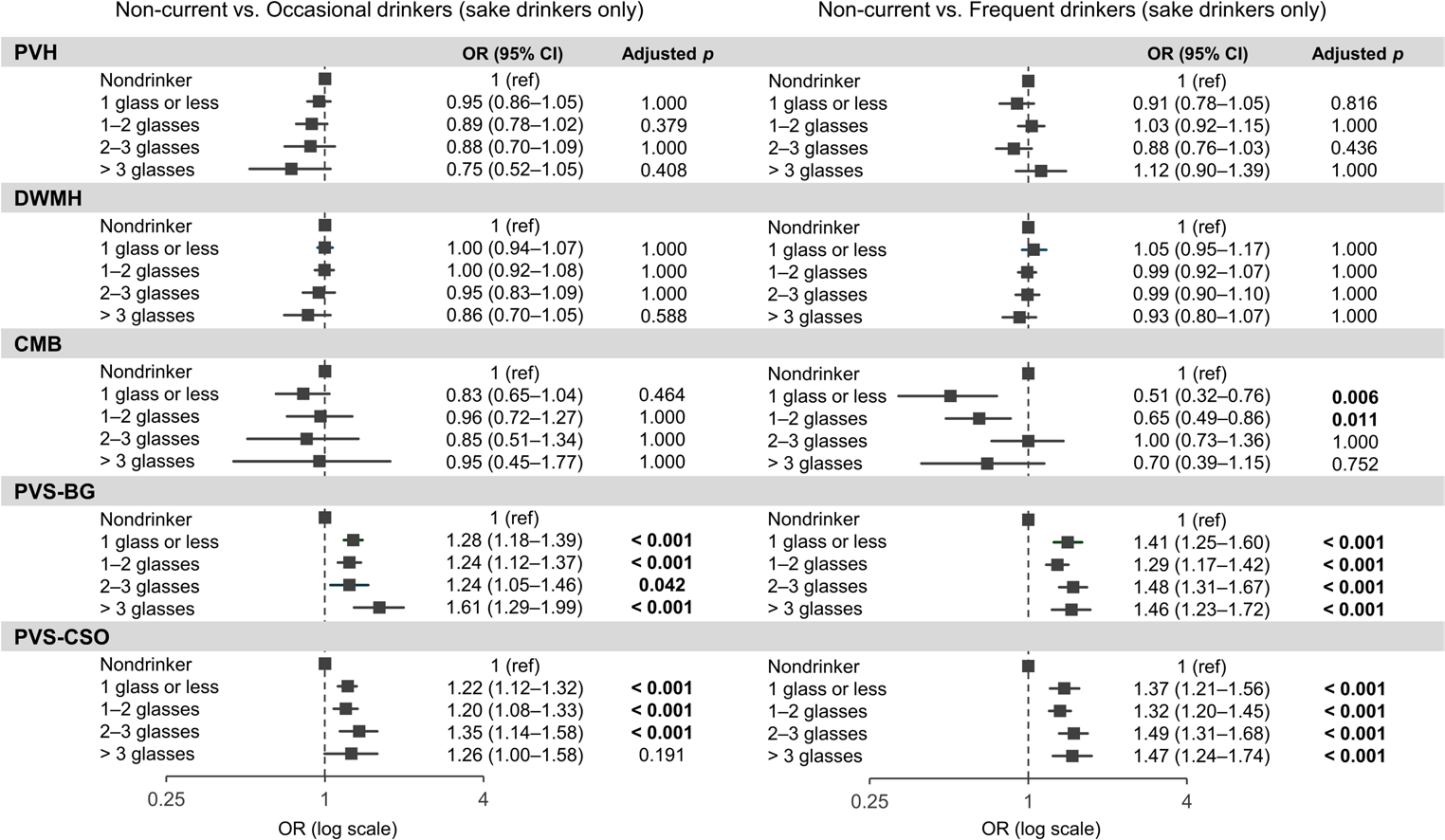
Forest plot summarizing the multivariable ordinal logistic regressions based on alcohol intake category among sake drinkers. All models were adjusted for age, sex, BMI, smoking habits, and histories of hypertension, diabetes mellitus, and dyslipidemia. The amount of alcohol consumed was expressed in units of sake, and the analyses did not include those who consumed alcohol other than sake. Bold values indicate alcohol intake categories that remained statistically significant after correction for multiple comparisons. PVH, periventricular hyperintensity; DWMH, deep subcortical white matter hyperintensity; CMB, cerebral microbleed; PVS, perivascular space; BG, basal ganglia; CSO, centrum semiovale; OR, odds ratio; CI, confidence interval; ref, reference

We further performed multivariable ordinal logistic regression analyses to examine whether the severity of SVD markers varied according to the amount of alcohol consumed, using non-current drinkers as the reference group (Fig. 4). The amount of alcohol consumed was measured in units of sake, and participants who consumed other types of alcohol were excluded from this analysis. After correction for multiple comparisons, significantly lower odds of CMB were observed in frequent drinkers consuming 1 glass or less (OR = 0.51, 95% CI: 0.32–0.76, adjusted *p* = 0.006) and 1–2 glasses (OR = 0.65, 95% CI: 0.49–0.86, adjusted *p* = 0.011). In contrast, consistently higher odds of PVS-BG and PVS-CSO was observed across nearly all alcohol intake categories among both occasional and frequent drinkers, with all adjusted *p* values < 0.001, except for the highest category of occasional drinking in PVS-CSO.

## Discussion

The present analysis revealed that habitual alcohol consumption was associated with greater severity of PVS enlargement. The association appeared moderate in magnitude but was consistent across drinking frequency categories. To the best of our knowledge, this is the first study to establish a clear link between habitual alcohol consumption and PVS enlargement in a relatively large cohort of community-dwelling adults. These findings provide novel evidence suggesting that habitual alcohol consumption may contribute to subtle structural alterations in the brain.

In the multivariable analysis, alcohol consumption was not statistically associated with PVH, DWMH, or CMB. However, subsequent interaction analyses revealed that occasional drinkers with lower BMI showed higher odds of PVH, whereas those with higher BMI exhibited lower odds of PVH. This seemingly paradoxical result may reflect the dual physiological effects of alcohol on the cerebrovascular system. Light-to-moderate alcohol consumption has been reported to lower the risk of ischemic stroke through its antihypertensive and antithrombotic effects [28, 29], whereas moderate-to-heavy alcohol intake can accelerate arteriosclerosis and promote brain atrophy [3, 30]. Thus, alcohol may either mitigate or exacerbate the risk of SVD markers depending on the level of alcohol exposure. One possible explanation for the reduced odds among individuals with higher BMI is that they may have a greater alcohol metabolism capacity and higher tolerance, rendering occasional alcohol consumption less harmful or even relatively protective in this subgroup. Furthermore, among frequent drinkers, alcohol intake up to two glasses of sake per day was significantly associated with reduced odds of CMB. Because CMB occurrence is more strongly related to individual vascular health status than to the normal aging process [31], these findings raise the possibility that moderate, regular alcohol consumption may confer a protective vascular effect, particularly against CMB formation. However, it should be noted that the present study did not distinguish the anatomical distribution of CMBs, and the effects of alcohol consumption may differ between lobar and deep CMBs. Further studies incorporating region-specific classification of CMBs are warranted to clarify whether alcohol consumption differentially affects these lesion types. Overall, the relationship between alcohol consumption and SVD markers is heterogeneous and may depend on the amount of alcohol consumed, individual characteristics such as BMI.

The association between alcohol consumption and PVS enlargement was relatively consistent across the subgroups, indicating that PVS enlargement was generally associated with alcohol consumption habits, regardless of the clinical characteristics. Although the observed odds ratios were modest in magnitude, they should be interpreted within the context of the ordinal outcome. Incremental increases in alcohol consumption frequency or amount were associated with a gradual upward shift in the distribution of PVS severity, suggesting a subtle but consistent influence on perivascular tissue integrity. Indeed, several studies have reported that PVS enlargement may reflect various physiological abnormalities in the brain tissue or cerebral vessels. For instance, disruption of the blood–brain barrier due to vascular endothelial dysfunction and spiral elongation of small arteries owing to atherosclerosis are considered important factors contributing to PVS enlargement [32]. Especially, PVS-BG is thought to be strongly influenced not only by aging but also by hypertension and smoking habits, suggesting that it may reflect a more pronounced manifestation of arteriosclerosis compared with PVS in other regions [33]. Another key factor associated with PVS enlargement is the reduction of surrounding brain volume and perivascular myelin loss [32]. Chronic alcohol consumption induces cortical and cerebellar atrophy and decreases CNS myelin, which may contribute to enlargement of PVS-CSO [3, 34]. Moreover, accumulating evidence indicates that PVS-CSO is more closely associated with amyloid-related pathology than with traditional vascular risk factors [35]. In this context, the stronger association observed between alcohol consumption and PVS-CSO in our study may reflect the influence of alcohol on neurodegenerative pathways rather than solely on vascular integrity. Taken together, our present findings suggest that PVS enlargement, particularly in the CSO, may reflect early neurodegenerative changes associated with habitual alcohol consumption.

In the current analysis, self-reported data were collected from participants who habitually drank sake to obtain a more accurate measurement of alcohol consumption. The multivariable ordinal logistic analyses stratified based on drinking frequency and sake intake showed that PVS-BG and PVS-CSO are moderately associated with habitual drinking. The discrepancy in ORs for PVS-BG between the results of the analysis including all occasional drinkers and the analysis restricted to sake drinkers may be owing to the fact that the occasional drinker group included many social drinkers who consumed significantly smaller amounts of beer or wine, far below the equivalent of one glass of sake. Notably, even among habitual drinkers who consumed approximately one glass of sake (equivalent to approximately 20 g of ethanol), the ORs for PVS-BG and PVS-CSO were similar to those who consumed more than one glass. In Japan, the recommended optimal level of alcohol consumption is approximately 20 g of ethanol per day, based on domestic and international follow-up studies [36, 37]. Our analysis suggests that habitual drinking at this recommended level may be associated with PVS enlargement. From a preventive perspective, reconsidering the definition of optimal alcohol intake and promoting balanced drinking habits may contribute to more effective dementia prevention strategies.

In this study, major lifestyle-related diseases were included as covariates because they could act as potential confounders in the association between alcohol consumption and SVD markers. Nevertheless, residual confounding cannot be fully excluded. For instance, higher alcohol intake has been identified as a risk factor for obstructive sleep apnea [38], which itself may promote the progression of SVD markers through intermittent hypoxia and vascular dysfunction. Furthermore, individual socioeconomic and educational status may also influence this relationship. Previous studies have reported that individuals with lower socioeconomic status are more likely to develop alcohol-related diseases even at comparable levels of alcohol consumption, and that minority or lower-education groups tend to experience greater alcohol-related harm [39, 40]. Future studies should therefore investigate the role of these unmeasured confounding factors in the relationship between alcohol exposure and cerebral small-vessel pathology in more detail.

The strength of this study lies in the multivariable analysis of a large cohort of adults undergoing brain health checkups. The models revealed that higher frequency of alcohol consumption was significantly associated with PVS enlargement. However, this study has some limitations. First, this was a cross-sectional study, which prevented the establishment of a causal association between alcohol consumption and cerebral SVD. Second, this study was based on data from a single facility in Japan, and the results may not be generalizable to racial or ethnic groups. This is because Asians generally have a lower capacity to metabolize alcohol compared with Western populations [41]. In addition, participants of the Brain Dock program are generally self-selected, health-conscious individuals who voluntarily undergo screening, and heavy drinkers may therefore be underrepresented in our cohort. This self-selection bias should be considered when interpreting the generalizability of our results. Third, because information on past alcohol consumption was unavailable, former drinkers were included in the non-current drinker group. This may have introduced bias due to the so-called “sick-quitter” effect, whereby individuals who stopped drinking because of health concerns were misclassified as non-current drinkers. The absence of data on drinking duration also implies that total alcohol exposure might have varied considerably even within the same drinking category, which may have introduced heterogeneity within categories and affected the robustness of our conclusions. Fourth, for the sake of efficacy in data collection, alcohol consumption data were limited to sake drinkers. Therefore, it remains unclear whether similar associations exist for other types of alcoholic beverages, such as beer and wine. Finally, MRI images were obtained using 1.5 T equipment, and the WML, CMB, and PVS were evaluated semiquantitatively by experts. Because 1.5 T MRI has lower spatial resolution than 3 T systems, smaller or less conspicuous lesions may have been missed or underestimated. Recently, automated methods for measuring the PVS volume have been proposed [42]. To achieve more detailed assessments of the PVS, future studies should use higher-field MRI and automated detection tools.

## Conclusions

We investigated the association between alcohol consumption habits, including sake intake, and cerebral SVD markers in a large, single-center cohort. Our findings indicate that PVS, particularly PVS-CSO, shows a moderate association with habitual alcohol use. These results suggest that PVS may serve as a potential imaging biomarker reflecting subtle structural brain alterations linked to alcohol exposure.

## Supplementary Information

Below is the link to the electronic supplementary material.

Supplementary Material 1 (DOCX 57.2 KB)

## Data Availability

The datasets generated during and/or analyzed during the current study are not publicly available due to ethical restrictions but are available from the corresponding author on reasonable request.

## References

[1] Rehm J, Gmel GE Sr., Gmel G et al (2017) The relationship between different dimensions of alcohol use and the burden of disease-an update. Addiction 112:968–1001. 10.1111/add.13757 28220587 10.1111/add.13757 PMC5434904

[2] Andersson C, Schou M, Gustafsson F et al (2022) Alcohol intake in patients with cardiomyopathy and heart failure: consensus and controversy. Circ Heart Fail 15:e009459. 10.1161/CIRCHEARTFAILURE.121.009459 35593142 10.1161/CIRCHEARTFAILURE.121.009459

[3] de la Monte SM, Kril JJ (2014) Human alcohol-related neuropathology. Acta Neuropathol 127:71–90. 10.1007/s00401-013-1233-3 24370929 10.1007/s00401-013-1233-3 PMC4532397

[4] Vetreno RP, Hall JM, Savage LM (2011) Alcohol-related amnesia and dementia: animal models have revealed the contributions of different etiological factors on neuropathology, neurochemical dysfunction and cognitive impairment. Neurobiol Learn Mem 96:596–608. 10.1016/j.nlm.2011.01.003 21256970 10.1016/j.nlm.2011.01.003 PMC3086968

[5] He X, Sullivan EV, Stankovic RK et al (2007) Interaction of thiamine deficiency and voluntary alcohol consumption disrupts rat corpus callosum ultrastructure. Neuropsychopharmacology 32:2207–2216. 10.1038/sj.npp.1301332 17299515 10.1038/sj.npp.1301332

[6] Koch M, Fitzpatrick AL, Rapp SR et al (2019) Alcohol consumption and risk of dementia and cognitive decline among older adults with or without mild cognitive impairment. JAMA Netw Open 2:e1910319. 10.1001/jamanetworkopen.2019.10319 31560382 10.1001/jamanetworkopen.2019.10319 PMC6777245

[7] Mukamal KJ, Kuller LH, Fitzpatrick AL et al (2003) Prospective study of alcohol consumption and risk of dementia in older adults. JAMA 289:1405–1413. 10.1001/jama.289.11.1405 12636463 10.1001/jama.289.11.1405

[8] Reynolds K, Lewis B, Nolen JD et al (2003) Alcohol consumption and risk of stroke: a meta-analysis. JAMA 289:579–588. 10.1001/jama.289.5.579 12578491 10.1001/jama.289.5.579

[9] Fein G, Shimotsu R, Di Sclafani V et al (2009) Increased white matter signal hyperintensities in long-term abstinent alcoholics compared with nonalcoholic controls. Alcohol Clin Exp Res 33:70–78. 10.1111/j.1530-0277.2008.00812.x 18976350 10.1111/j.1530-0277.2008.00812.x PMC2629790

[10] Duering M, Biessels GJ, Brodtmann A et al (2023) Neuroimaging standards for research into small vessel disease-advances since 2013. Lancet Neurol 22:602–618. 10.1016/S1474-4422(23)00131-X 37236211 10.1016/S1474-4422(23)00131-X

[11] Libecap TJ, Pappas CA, Bauer CE et al (2024) Enlarged perivascular space burden predicts declines in cognitive and functional performance. J Neurol Sci 466:123232. 10.1016/j.jns.2024.123232 39298972 10.1016/j.jns.2024.123232 PMC11563846

[12] Jeong SH, Cha J, Park M et al (2022) Association of enlarged perivascular spaces with amyloid burden and cognitive decline in alzheimer disease continuum. Neurology 99:e1791–e1802. 10.1212/WNL.0000000000200989 35985826 10.1212/WNL.0000000000200989

[13] Liu H, Meng L, Wang J et al (2024) Enlarged perivascular spaces in alcohol-related brain damage induced by dyslipidemia. J Cereb Blood Flow Metab 44:1867–1880. 10.1177/0271678X241251570 38700501 10.1177/0271678X241251570 PMC11494831

[14] Ren Y, Meng K, Sun Y et al (2023) Effects of white matter lesion grading on the cognitive function of patients with chronic alcohol dependence. Am J Transl Res 15:1129–1139. https://www.ncbi.nlm.nih.gov/pubmed/36915744 36915744 PMC10006824

[15] Morita A (2019) Value of brain dock (Brain Screening) system in Japan. World Neurosurg 127:502. 10.1016/j.wneu.2019.04.211 31048044 10.1016/j.wneu.2019.04.211

[16] Masuo Y, Imai T, Shibato J et al (2009) Omic analyses unravels global molecular changes in the brain and liver of a rat model for chronic sake (Japanese alcoholic beverage) intake. Electrophoresis 30:1259–1275. 10.1002/elps.200900045 19382137 10.1002/elps.200900045

[17] Fazekas F, Chawluk JB, Alavi A et al (1987) MR signal abnormalities at 1.5 T in Alzheimer’s dementia and normal aging. AJR Am J Roentgenol 149:351–356. 10.2214/ajr.149.2.351 3496763 10.2214/ajr.149.2.351

[18] Shinohara Y, Tohgi H, Hirai S et al (2007) Effect of the Ca antagonist nilvadipine on stroke occurrence or recurrence and extension of asymptomatic cerebral infarction in hypertensive patients with or without history of stroke (PICA Study). 1. Design and results at enrollment. Cerebrovasc Dis 24:202–209. 10.1159/000104478 17596689 10.1159/000104478

[19] Katayama Y (2019) The Japan brain dock society guideline 2019 (in Japanese). Kyoubunnsya, Hokkaido, Japan

[20] Gons RA, van Norden AG, de Laat KF et al (2011) Cigarette smoking is associated with reduced microstructural integrity of cerebral white matter. Brain 134:2116–2124. 10.1093/brain/awr145 21705426 10.1093/brain/awr145

[21] Omori N, Ikawa F, Chiku M et al (2024) Dose-dependent effect of current smoking on enlarged perivascular space identified on brain magnetic resonance imaging. Cerebrovasc Dis. 10.1159/000541657 39348801 10.1159/000541657

[22] Friedman JI, Tang CY, de Haas HJ et al (2014) Brain imaging changes associated with risk factors for cardiovascular and cerebrovascular disease in asymptomatic patients. JACC Cardiovasc Imaging 7:1039–1053. 10.1016/j.jcmg.2014.06.014 25323165 10.1016/j.jcmg.2014.06.014

[23] Koennecke HC (2006) Cerebral microbleeds on MRI: prevalence, associations, and potential clinical implications. Neurology 66:165–171. 10.1212/01.wnl.0000194266.55694.1e 16434647 10.1212/01.wnl.0000194266.55694.1e

[24] Sugai Y, Hiraka T, Shibata A et al (2024) The time-course augmentation of perivascular space enlargement in the basal ganglia among a community-dwelling elder population. Jpn J Radiol 42:1110–1121. 10.1007/s11604-024-01595-3 38896331 10.1007/s11604-024-01595-3 PMC11442546

[25] Martinez-Ramirez S, Pontes-Neto OM, Dumas AP et al (2013) Topography of dilated perivascular spaces in subjects from a memory clinic cohort. Neurology 80:1551–1556. 10.1212/WNL.0b013e31828f1876 23553482 10.1212/WNL.0b013e31828f1876 PMC3662325

[26] Ogawa W, Hirota Y, Miyazaki S et al (2024) Definition, criteria, and core concepts of guidelines for the management of obesity disease in Japan. Endocr J 71:223–231. 10.1507/endocrj.EJ23-0593 38123337 10.1507/endocrj.EJ23-0593

[27] Kanda Y (2013) Investigation of the freely available easy-to-use software ‘EZR’ for medical statistics. Bone Marrow Transpl 48:452–458. 10.1038/bmt.2012.244 10.1038/bmt.2012.244 PMC3590441 23208313

[28] Song RJ, Larson MG, Aparicio HJ et al (2023) Moderate alcohol consumption on the risk of stroke in the million veteran program. BMC Public Health 23:2485. 10.1186/s12889-023-17377-x 38087273 10.1186/s12889-023-17377-x PMC10714616

[29] Minzer S, Losno RA, Casas R (2020) The effect of alcohol on cardiovascular risk factors: is there new information? Nutrients. 10.3390/nu12040912 32230720 10.3390/nu12040912 PMC7230699

[30] Zakhari S (1997) Alcohol and the cardiovascular system: molecular mechanisms for beneficial and harmful action. Alcohol Health Res World 21:21–29. https://www.ncbi.nlm.nih.gov/pubmed/15706760 15706760 PMC6826791

[31] Daugherty AM, Raz N (2017) Incident risk and progression of cerebral microbleeds in healthy adults: a multi-occasion longitudinal study. Neurobiol Aging 59:22–29. 10.1016/j.neurobiolaging.2017.07.003 28800410 10.1016/j.neurobiolaging.2017.07.003 PMC5612885

[32] Okar SV, Hu F, Shinohara RT et al (2023) The etiology and evolution of magnetic resonance imaging-visible perivascular spaces: systematic review and meta-analysis. Front Neurosci 17:1038011. 10.3389/fnins.2023.1038011 37065926 10.3389/fnins.2023.1038011 PMC10098201

[33] Lara FR, Scruton AL, Pinheiro A et al (2022) Aging, prevalence and risk factors of MRI-visible enlarged perivascular spaces. Aging 14:6844–6858. 10.18632/aging.204181 35852852 10.18632/aging.204181 PMC9512514

[34] Rice J, Gu C (2019) Function and mechanism of Myelin regulation in alcohol abuse and alcoholism. BioEssays 41:e1800255. 10.1002/bies.201800255 31094014 10.1002/bies.201800255 PMC6597285

[35] Charidimou A, Boulouis G, Frosch MP et al (2022) The Boston criteria version 2.0 for cerebral amyloid angiopathy: a multicentre, retrospective, MRI-neuropathology diagnostic accuracy study. Lancet Neurol 21:714–725. 10.1016/S1474-4422(22)00208-3 35841910 10.1016/S1474-4422(22)00208-3 PMC9389452

[36] Tsugane S, Fahey MT, Sasaki S et al (1999) Alcohol consumption and all-cause and cancer mortality among middle-aged Japanese men: seven-year follow-up of the JPHC study cohort I. Japan public health center. Am J Epidemiol 150:1201–1207. 10.1093/oxfordjournals.aje.a009946 10588080 10.1093/oxfordjournals.aje.a009946

[37] Holman CD, English DR, Milne E et al (1996) Meta-analysis of alcohol and all-cause mortality: a validation of NHMRC recommendations. Med J Aust 164:141–145. 10.5694/j.1326-5377.1996.tb122011.x 8628131 10.5694/j.1326-5377.1996.tb122011.x

[38] Simou E, Britton J, Leonardi-Bee J (2018) Alcohol and the risk of sleep apnoea: a systematic review and meta-analysis. Sleep Med 42:38–46. 10.1016/j.sleep.2017.12.005 29458744 10.1016/j.sleep.2017.12.005 PMC5840512

[39] Edwards AC, Larsson Lonn S, Chartier KG et al (2024) Socioeconomic position indicators and risk of alcohol-related medical conditions: A National cohort study from Sweden. PLoS Med 21:e1004359. 10.1371/journal.pmed.1004359 38502640 10.1371/journal.pmed.1004359 PMC10950249

[40] Collins SE (2016) Associations between socioeconomic factors and alcohol outcomes. Alcohol Res 38:83–94. https://www.ncbi.nlm.nih.gov/pubmed/27159815 27159815 10.35946/arcr.v38.1.11 PMC4872618

[41] Edenberg HJ, Foroud T (2006) The genetics of alcoholism: identifying specific genes through family studies. Addict Biol 11:386–396. 10.1111/j.1369-1600.2006.00035.x 16961766 10.1111/j.1369-1600.2006.00035.x

[42] Pham W, Lynch M, Spitz G et al (2022) A critical guide to the automated quantification of perivascular spaces in magnetic resonance imaging. Front Neurosci 16:1021311. 10.3389/fnins.2022.1021311 36590285 10.3389/fnins.2022.1021311 PMC9795229

