# Supplementary material for: Enlargement of perivascular spaces associated with habitual sake (Japanese rice wine) consumption in participants of brain health checkups
Source: Neuroradiology. 2026 Feb 6;68(7):1767–76. doi: 10.1007/s00234-026-03917-w (PMC13407583; doi:10.1007/s00234-026-03917-w)
Supplement: Supplementary file 1 — Supplementary Material 1 (DOCX 57.2 KB) [file 234_2026_3917_MOESM1_ESM.docx]

**Supplementary materials**

**Data collection**

The dataset used in this study was obtained from the Brain Dock database, a brain screening service provided by EUCALIA Inc. This service was launched in 2018 with the aim of detecting asymptomatic cerebral abnormalities such as unruptured aneurysms and silent cerebral infarctions at an early stage using brain magnetic resonance imaging (MRI) and magnetic resonance angiography. Users can make appointments and complete pre-examination questionnaires online and receive their results via computer or smartphone. As a result, the average clinic visit time is reduced to approximately 30 minutes, providing a rapid and efficient screening process. The dataset for this study was collected from participants who underwent brain health checkups at the Medical Check Studio Tokyo Ginza from January 2018 to December 2024. All data were centrally managed by the clinic’s supervising physician, and the measurement protocol remained consistent throughout the study period.

**Clinical assessment**

Clinical data of the participants were collected through an online pre-examination questionnaire completed by the participants themselves and through physical measurements taken on the day of MRI scanning. Participants were asked about their medical histories, including hypertension, diabetes mellitus, and dyslipidemia. These conditions were considered present if the participant was receiving treatment for these conditions or had been diagnosed by a medical specialist.

In Japan, these conditions are diagnosed in accordance with national clinical practice guidelines. Specifically:

1. Hypertension was defined as a systolic blood pressure ≥ 140 mmHg and/or diastolic blood pressure ≥ 90 mmHg, based on the Japanese Society of Hypertension Guidelines.[1]
2. Diabetes mellitus was defined according to the diagnostic criteria of the Japan Diabetes Society.[2] Patients were considered to have diabetes mellitus if they met the diagnostic thresholds for diabetes on two separate occasions, including a fasting plasma glucose level ≥ 126 mg/dL, a 2 hour post-load glucose ≥ 200 mg/dL during a 75 g oral glucose tolerance test, or an hemoglobin (Hb) A1c level ≥ 6.5%. A single abnormal test result was not sufficient for diagnosis in the absence of clinical symptoms; instead, a repeated test on another day was required for a definitive diagnosis.
3. Dyslipidemia was defined as low-density lipoprotein (LDL) cholesterol ≥ 140 mg/dL, high-density-lipoprotein (HDL) cholesterol < 40 mg/dL, triglycerides ≥ 150 mg/dL, in accordance with the Japan Atherosclerosis Society Guidelines.[3]

On the day of the examination, clinic staff reviewed the questionnaire responses with each participant to confirm the accuracy of the reported medical histories, smoking habits, and alcohol consumption habits.

**Neuroimaging**

All MR images were reviewed through a double-reading system conducted by board-certified physicians. Initially, the images obtained from Brain Dock were uploaded to a secure cloud-based platform. The first reading was performed by one of 15 board-certified neuroradiologists, followed by a second reading conducted by one of 14 board-certified neurosurgeons. Importantly, the clinical information of the participants was blinded to the physicians during the image interpretation. Although formal inter- or intra-rater reliability analyses (e.g., κ or ICC) were not conducted, any discrepancies between readers were resolved through consensus discussion to ensure consistency of ratings.

The assessment of cerebral small vessel diseases in this study was conducted in accordance with the Japanese Brain Dock Guidelines.[4] These guidelines provide detailed criteria for evaluating the severity of MRI findings related to cerebral small vessel disease. For example, the severity of **periventricular hyperintensity (PVH)** and **deep white matter hyperintensity (DWMH)** is graded on a scale from 0 to IV, based on criteria adapted from Fazekas et al.[5]

PVH was classified as follows:

Grade 0, absent or “periventricular rims” only;

Grade I, localized lesion “cap” or pencil-thin lining;

Grade II, extended along the entire periventricular area;

Grade III, irregular PVH extending into the deep white matter;

Grade IV, extending throughout the deep and subcortical white matter.

DWMH was classified as follows:

Grade 0, absent;

Grade I, punctate foci, diameter ≤ 3 mm, boundary clear;

Grade II, diameter ≥ 3 mm, punctate or discrete foci;

Grade III, large confluent foci, boundary unclear;

Grade IV, confluence widely distributed in most of the white matter.

In addition, **cerebral microbleeds (CMB)** and **perivascular spaces (PVS)** were evaluated based on lesion counts.

CMBs are small (generally 2–5 mm diameter) areas of signal void with blooming artifacts on T2*-weighted MRI, and were classified as follows:

Grade 0, none;

Grade I counts 1 lesion;

Grade II, counts 2–5 lesions;

Grade III, counts 6–10 lesions;

Grade IV, counts ≥ 11 lesions

PVSs were defined as ovoid or linear lesions that were visible as hypointense and hyperintense region in the basal ganglia (PVS-BG) or centrum semiovale (PVS-CSO) on T1 and T2-weighted images, respectively, and were considered “enlarged” if their size was ≥ 2 mm.

PVS-BG was graded as follows (0–III):

Grade 0, none;

Grade I, counts 1–5 lesions;

Grade II, counts 6–10 lesions;

Grade III, counts ≥ 11 lesions.

PVS-CSO was graded as follows (0–IV):

Grade 0, none;

Grade I, counts 1–10 lesions;

Grade II, counts 11–20 lesions;

Grade III, counts 21–40 lesions;

Grade IV, counts ≥ 41 lesions.

**Supplementary Table 1 MRI scan protocol and details used in this study.**

| Manufacturer name | CANON | | | |
| --- | --- | --- | --- | --- |
| Model name | Vantage Elan | | | |
| Sequence | T1 | T2 | FLAIR | T2* |
| Protocol | SE | FSE | FSE | GRE |
| TR (ms) | 480 | 4100 | 10000 | 520 |
| TE (ms) | 10 | 100 | 110 | 13.5 |
| FOV (mm) | 230 | 230 | 230 | 230 |
| Matrix | 160×256 | 224×320 | 192×256 | 176×288 |
| Slice thickness (mm) | 6 | 6 | 6 | 1.2 |
| Interslice gap (mm) | 1.2 | 1.2 | 1.2 | 1.2 |
| Number of slices | 20 | 20 | 20 | 20 |
| Imaging time (min) | 0：47 | 0：29 | 1：40 | 0：47 |

MRI, magnetic resonance imaging; FLAIR, fluid-attenuated inversion recovery; SE, spin echo; FSE, fast spin echo; GRE, gradient echo; TR, repetition time; TE, echo time; FOV, field of view.

**Supplementary Table 2 Severity of cerebral small vessel diseases stratified by alcohol consumption habits.**

|  | **Total** | **Non-current drinkers** | **Occasional drinkers** | **Frequent drinkers** |
| --- | --- | --- | --- | --- |
|  | n = 64,659 | n = 19,671 | n = 25,582 | n = 19,406 |
| Asymptomatic lesions, n (%) |  |  |  |  |
| PVH |  |  |  |  |
| Grade 0 | 56,001 (86.6) | 16,791 (85.4) | 22,774 (89.0) | 16,436 (84.7) |
| Grade I | 7,584 (11.7) | 2,451 (12.5) | 2,496 (9.8) | 2,637 (13.6) |
| Grade II | 1,012 (1.6) | 402 (2.0) | 297 (1.2) | 313 (1.6) |
| Grade III | 58 (0.1) | 26 (0.1) | 14 (0.1) | 18 (0.1) |
| Grade IV | 4 (0.0) | 1 (0.0) | 1 (0.0) | 2 (0.0) |
| DSWMH |  |  |  |  |
| Grade 0 | 38,980 (60.3) | 11,660 (59.3) | 16,382 (64.0) | 10,938 (56.4) |
| Grade I | 21,470 (33.2) | 6,551 (33.3) | 7,824 (30.6) | 7,095 (36.6) |
| Grade II | 3,921 (6.1) | 1,324 (6.7) | 1,304 (5.1) | 1,293 (6.7) |
| Grade III | 285 (0.4) | 135 (0.7) | 71 (0.3) | 79 (0.4) |
| Grade IV | 3 (0.0) | 1 (0.0) | 1 (0.0) | 1 (0.0) |
| CMB |  |  |  |  |
| Grade 0 | 63360 (98.0) | 19252 (97.9) | 25131 (98.2) | 18977 (97.8) |
| Grade I | 969 (1.5) | 324 (1.6) | 332 (1.3) | 313 (1.6) |
| Grade II | 270 (0.4) | 78 (0.4) | 96 (0.4) | 96 (0.5) |
| Grade III | 42 (0.1) | 9 (0.0) | 19 (0.1) | 14 (0.1) |
| Grade IV | 18 (0.0) | 8 (0.0) | 4 (0.0) | 6 (0.0) |
| PVS-BG |  |  |  |  |
| Grade 0 | 54,420 (84.2) | 16,642 (84.6) | 21,878 (85.5) | 15,900 (81.9) |
| Grade I | 9,602 (14.8) | 2,831 (14.4) | 3,506 (13.7) | 3,265 (16.8) |
| Grade II | 552 (0.9) | 165 (0.8) | 173 (0.7) | 214 (1.1) |
| Grade III | 85 (0.1) | 33 (0.2) | 25 (0.1) | 27 (0.1) |
| PVS-CSO |  |  |  |  |
| Grade 0 | 54,674 (84.6) | 16,946 (86.2) | 21,895 (85.6) | 15,833 (81.6) |
| Grade I | 8,887 (13.7) | 2,434 (12.3) | 3,316 (13.0) | 3,137 (16.2) |
| Grade II | 1,000 (1.5) | 260 (1.3) | 341 (1.3) | 399 (2.1) |
| Grade III | 94 (0.1) | 28 (0.1) | 30 (0.1) | 36 (0.1) |
| Grade IV | 3 (0.0) | 2 (0.0) | 0 (0.0) | 1 (0.0) |

The severity was classified from grades 0 to IV for PVH, DWMH, CMB, and PVS-CSO. For PVS-BG, severity was classified from grades 0 to III. PVH, periventricular hyperintensity; DWMH, deep subcortical white matter hyperintensity; CMB, cerebral microbleed; PVS, perivascular space; BG, basal ganglia; CSO, centrum semiovale.

**Supplementary Table 3 Variance inflation factors and Brant test results from ordinal logistic regression models.**

| Variables | Non-current vs. Occasional drinkers | | Non-current vs. Frequent drinkers | |
| --- | --- | --- | --- | --- |
|  | VIF | Brant test (*p* value) | VIF | Brant test (*p* value) |
| PVH |  |  |  |  |
| Age | 1.08 | < 0.001 | 1.03 | < 0.001 |
| Sex | 1.23 | 0.325 | 1.17 | 0.093 |
| BMI | 1.21 | 0.617 | 1.10 | 0.383 |
| Smoking habits | 1.02 |  | 1.03 |  |
| Nonsmoker |  | Ref |  | Ref |
| Former smoker |  | 0.563 |  | 0.858 |
| Current smoker |  | 0.757 |  | 0.895 |
| Exposure to secondhand smoke |  | 0.700 |  | 0.981 |
| Hypertension | 1.15 | 0.038 | 1.07 | 0.004 |
| Diabetes mellitus | 1.08 | 0.937 | 1.03 | 0.996 |
| Dyslipidemia | 1.04 | 0.879 | 1.02 | 0.492 |
| Alcohol consumption | 1.05 | 0.510 | 1.10 | 0.086 |
| DWMH |  |  |  |  |
| Age | 1.09 | < 0.001 | 1.04 | < 0.001 |
| Sex | 1.10 | 0.015 | 1.15 | 0.001 |
| BMI | 1.11 | 0.218 | 1.10 | 0.153 |
| Smoking habits | 1.02 |  | 1.03 |  |
| Nonsmoker |  | Ref |  | Ref |
| Former smoker |  | 0.458 |  | 0.054 |
| Current smoker |  | 0.846 |  | 0.739 |
| Exposure to secondhand smoke |  | 0.898 |  | 0.837 |
| Hypertension | 1.07 | < 0.001 | 1.07 | < 0.001 |
| Diabetes mellitus | 1.04 | 0.180 | 1.03 | 0.760 |
| Dyslipidemia | 1.03 | 0.992 | 1.03 | 0.137 |
| Alcohol consumption | 1.02 | 0.001 | 1.08 | 0.031 |
| CMB |  |  |  |  |
| Age | 1.09 | 0.045 | 1.07 | < 0.001 |
| Sex | 1.10 | 0.155 | 1.15 | 0.216 |
| BMI | 1.10 | 0.911 | 1.09 | 0.750 |
| Smoking habits | 1.02 |  | 1.03 |  |
| Nonsmoker |  | Ref |  | Ref |
| Former smoker |  | 0.820 |  | 0.157 |
| Current smoker |  | 0.290 |  | 0.005 |
| Exposure to secondhand smoke |  | 0.997 |  | 0.245 |
| Hypertension | 1.11 | 0.024 | 1.10 | 0.022 |
| Diabetes mellitus | 1.05 | 0.672 | 1.03 | 0.916 |
| Dyslipidemia | 1.02 | 0.476 | 1.02 | 0.549 |
| Alcohol consumption | 1.03 | 0.141 | 1.10 | 0.197 |
| PVS-BG |  |  |  |  |
| Age | 1.07 | < 0.001 | 1.06 | < 0.001 |
| Sex | 1.10 | 0.001 | 1.14 | 0.003 |
| BMI | 1.10 | 0.309 | 1.10 | 0.624 |
| Smoking habits | 1.01 |  | 1.03 |  |
| Nonsmoker |  | Ref |  | Ref |
| Former smoker |  | 0.237 |  | 0.101 |
| Current smoker |  | 0.772 |  | 0.703 |
| Exposure to secondhand smoke |  | 0.761 |  | 0.751 |
| Hypertension | 1.09 | < 0.001 | 1.09 | 0.035 |
| Diabetes mellitus | 1.04 | 0.481 | 1.03 | 0.074 |
| Dyslipidemia | 1.03 | 0.308 | 1.03 | 0.611 |
| Alcohol consumption | 1.02 | 0.844 | 1.08 | 0.178 |
| PVS-CSO |  |  |  |  |
| Age | 1.08 | < 0.001 | 1.07 | < 0.001 |
| Sex | 1.09 | 0.003 | 1.13 | < 0.001 |
| BMI | 1.10 | 0.120 | 1.10 | 0.280 |
| Smoking habits | 1.02 |  | 1.03 |  |
| Nonsmoker |  | Ref |  | Ref |
| Former smoker |  | 0.352 |  | 0.449 |
| Current smoker |  | 0.789 |  | 0.242 |
| Exposure to secondhand smoke |  | 1.000 |  | 1.000 |
| Hypertension | 1.09 | 0.244 | 1.09 | 0.887 |
| Diabetes mellitus | 1.04 | 0.826 | 1.03 | 0.228 |
| Dyslipidemia | 1.03 | 0.265 | 1.03 | 0.774 |
| Alcohol consumption | 1.02 | 0.699 | 1.08 | 0.633 |

VIF, Variance inflation factors; PVH, periventricular hyperintensity; DWMH, deep subcortical white matter hyperintensity; CMB, cerebral microbleeds; PVS, perivascular space; BG, basal ganglia; CSO, centrum semiovale; BMI, body mass index; Ref, reference.

**References**

[1] Umemura S, Arima H, Arima S, et al. The Japanese Society of Hypertension Guidelines for the Management of Hypertension (JSH 2019). Hypertens Res. 2019;42:1235–481. <https://doi.org/10.1038/s41440-019-0284-9>

[2] Araki E, Goto A, Kondo T, et al. Japanese Clinical Practice Guideline for Diabetes 2019. J Diabetes Investig. 2020;11:1020–76. <https://doi.org/10.1111/jdi.13306>

[3] Kinoshita M, Yokote K, Arai H, et al. Japan Atherosclerosis Society (JAS) Guidelines for Prevention of Atherosclerotic Cardiovascular Diseases 2017. J Atheroscler Thromb. 2018;25:846–984. <https://doi.org/10.5551/jat.GL2017>

[4] Katayama Y. The Japan Brain Dock society guideline 2019 (in Japanese). Hokkaido, Japan: Kyoubunnsya; 2019.

[5] Fazekas F, Chawluk JB, Alavi A, et al. MR signal abnormalities at 1.5 T in Alzheimer's dementia and normal aging. AJR Am J Roentgenol. 1987;149:351–6. <https://doi.org/10.2214/ajr.149.2.351>
